# Highly thermostable RhB@Zr-Eddc for the selective sensing of nitrofurazone and efficient white light emitting diode

**DOI:** 10.3389/fchem.2024.1444036

**Published:** 2024-08-02

**Authors:** Yanqiong Shen, Di Ma, Mian Zhao, Jinjie Qian, Qipeng Li

**Affiliations:** ^1^ College of Chemistry and Chemical Engineering, Zhaotong University, Zhaotong, China; ^2^ Experimental Center for Teaching, Hebei Medical University, Shijiazhuang, China; ^3^ College of Chemistry and Materials Engineering, Wenzhou University, Wenzhou, China

**Keywords:** RhB@Zr-Eddc, highly thermostable, nitrofurazone, selective detection, WLED

## Abstract

Highly thermostable **RhB@Zr-Eddc** composites with the Rhodamine B (**RhB**) enclosed into the nanocages of **Zr-Eddc** was synthesized by one-pot method under hydrothermal conditions, whose structure, morphology and stability were characterized through the X-ray powder diffractometry (XRD), scanning electron microscopy (SEM) and thermogravimetric analysis (TGA). **RhB@Zr-Eddc** showed the highly thermal stability up to 550°C and emitted the bright red-light emission at 605 nm, which could highly selective detect the nitrofurazone (NFZ) among eleven other antibiotics in aqueous solution. Furthermore, via combining the **RhB@Zr-Eddc** with commercial green phosphor (Y_3_Al_5_O_12_:Ce^3+^, Ga^3+^), the mixture was encapsulated onto a 455 nm blue LED chip, creating an ex-cellent white light emitting diode (WLED) device with the correlated colour temperature (CCT) of 4710 K, luminous efficiency (LE) of 43.17 lm/w and Color Rendering Index (CRI) of 89.2.

## 1 Introduction

With the rapid expansion of livestock farming and aquaculture on a large scale, antibiotic veterinary drugs are extensively employed as feed additives to enhance animal growth and prevent/treat diseases in livestock and poultry (Hutchings et al., 2019; Svetlana et al., 2020; Divysnshi et al., 2022). Global statistics indicate an annual consumption of veterinary antibiotics exceeding 63,000 tons in areas like animal husbandry and aquaculture. This data is projected to rise to 106,600 tons by 2030, with quinolones, tetracyclines, sulphonamides, and others being the most commonly used veterinary drugs (Pepper et al., 2018; Kovalakova et al., 2020; Lyu et al., 2020). Due to the lack of effective regulation, a significant quantity of antibiotic-based veterinary drugs is consumed by animals. This results in enriched residues found in animal tissues, organs, and edible products, while livestock excretions and aquaculture wastewater discharge into the environment, causing environmental pollution and posing significant risks to human health (Baaloudj et al., 2021). Furthermore, due to the poor absorption of antibiotic veterinary drugs by organisms, these residues can persist in soil and water bodies, leading to microbial flora drug resistance and various ecological issues (Zhang et al., 2015; Chen et al., 2022; Zhang et al., 2024). This situation has a profound impact on human health and disrupts ecological balance. The complexity of antibiotic veterinary drug structures and the technical challenges in their detection make fluorescence detection methods, particularly those employing fluorescent sensors and signals, crucial. These methods offer high sensitivity, rapid response, and simplicity in operation, making them widely adopted in sensing detection (Chen et al., 2017; Zhou et al., 2018; Saboor et al., 2022). Given these considerations, the development of an easily operable, cost-effective and simple fluorescent sensor for detecting antibiotic residues is paramount for ensuring human health and environmental safety.

Metal-Organic Frameworks (MOFs) constitute a class of functional materials formed through the self-assembly of metal ions or clusters with organic ligands via ligand bonding. Among these, MOFs with fluorescent properties combine the stability of MOFs with high fluorescence efficiency. This unique combination allows for efficient fluorescence sensing through fluorescence bursts, rendering them highly applicable in the detection of antibiotic and pesticide residues (Hou et al., 2018; Zhu et al., 2018; Zhao et al., 2019; Duan et al., 2020; Yin et al., 2023). Despite their versatility, there is a scarcity of fluorescent MOFs-based composites exhibiting both high selectivity and sensitivity in the detection of antibiotics (Wang et al., 2022; Heng et al., 2023; Li et al., 2023; Luo et al., 2023; Zhong et al., 2023; Lei et al., 2024; Wu et al., 2024). Consequently, there is an urgent need to design and prepare more MOFs-based fluorescent composites tailored for high-selectivity, high-sensitivity antibiotic detection.

In this paper, the red Rhodamine B (**RhB**) fluorescent dye into the nanopores or cages of **Zr-Eddc** material to create highly thermally stable and efficient **RhB@Zr-Eddc**. The aim is to explore their potential applications in antibiotic detection and as components in white light emitting diode (WLED) devices. The findings from this research are expected to offer both experimental and theoretical guidance for the future development of MOFs fluorescent composites specifically designed for antibiotic detection and WLED devices.

## 2 Results and discussion

### 2.1 Preparation of materials, structural characterization and their structural analysis

**FIGURE 1:**
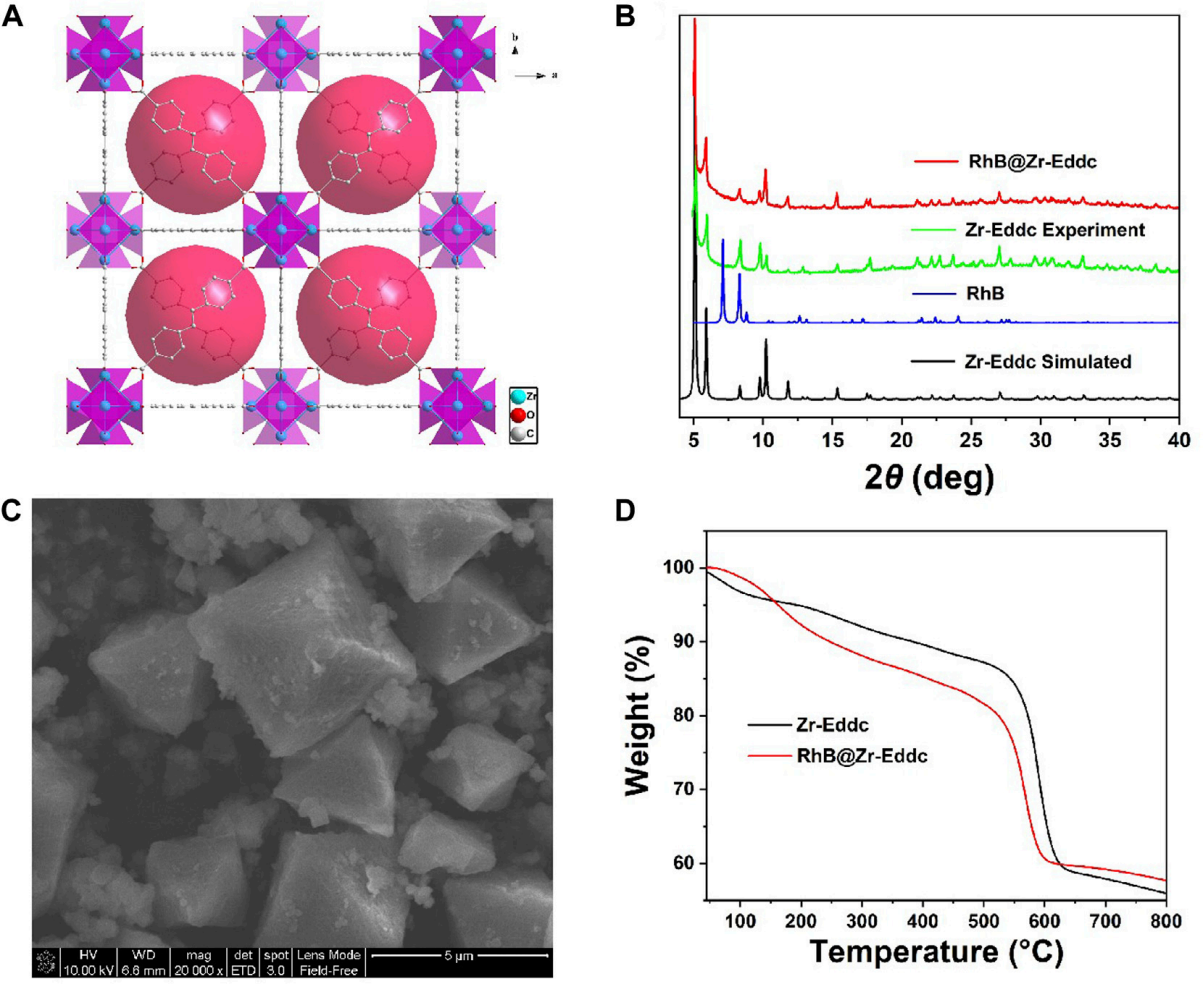
**(A)** Structure of the **RhB@Zr-Eddc**; **(B)** XRD powder diffraction diagram of **RhB** and **RhB@Zr-Eddc**; **(C)** SEM scanning diagram of **RhB@Zr-Eddc**; **(D)** Thermogravimetric curve of **RhB@Zr-Eddc**.

H_2_Eddc, ZrCl_4_ and RhB dye were utilized in the preparation of **RhB@Zr-Eddc** through one-pot method under hydrothermal conditions, where the red **RhB** dye was incorporated into the nano-cage of **Zr-Eddc** (Figure 1A and Supplementary Figure S2). Characterization of the prepared **Zr-Eddc** and **RhB@Zr-Eddc** was conducted using XRD, revealing that the XRD peaks of **RhB@Zr-Eddc** align well with those of **Zr-Eddc**, confirming the successful synthesis of both materials (Figure 1B). Thermogravimetric analysis (TGA) of **Zr-Eddc** primarily indicated the loss of guest solvent molecules up to 300°C, while **RhB@Zr-Eddc** lost both guest solvent molecules and **RhB** dye molecules until 300°C. The frameworks of both materials began to decompose from 550°C, highlighting the highly thermal stability of **Zr-Eddc** and **RhB@Zr-Eddc** (Figure 1D and Supplementary Figure S5A). Additionally, the morphology of **RhB@Zr-Eddc** was more intuitively observed through SEM, revealing generally regular octahedral particles, albeit with some variation in size (Figure 1C).

The structure of **Zr-Eddc** crystallizes in the cubic, and the linear carboxylates (*μ*_2_-COO) derived from the di H_2_Eddc ligand form the a Zr_6_O_4_(OH)_4_(CO_2_)_12_ cluster (Zhang et al., 2017). Structure of **Zr-Eddc** includes two types of polyhedral cages: an octahedral cage with a distance of 24.6 Å and tetrahedral cages with a distance of 17.4 Å (Supplementary Figure S1). The dimensions of the **RhB** dye molecule, measuring 15.6 Å × 13.5 Å × 4.2 Å (Supplementary Figure S3), are smaller than the nano-cage sizes of **Zr-Eddc** with the sizes of 24.6 Å and 17.4 Å. Structural analysis (Shen et al., 2022; Shen et al., 2023), as well as XRD data, support the conclusion that **RhB** dye has been encapsulated into the nano-cage of **Zr-Eddc** with the **RhB** dye concentration of 1.15 wt% (Supplementary Figure S4).

### 2.2 Analysis of the fluorescence properties

Due to **RhB@Zr-Eddc** was stable in conventional organic solvents and highly stable up to 550°C (Supplementary Figures S5A, B), the fluorescence emission wavelength of liquid-state **RhB@Zr-Eddc** was tested at room temperature with an excitation wavelength of 360 nm. **RhB@Zr-Eddc** exhibited a vibrant red-light emission with a maximum peak at 606 nm, primarily attributed to the characteristic emission of the **RhB** dye. To assess the potential of **RhB@Zr-Eddc** as a fluorescent probe for the detection of antibiotics, twelve different antibiotics, including furazolidone (FZD), nitrofurazone (NFZ), nitrofurantoin (NFT), ro-nidazole (RDZ), metronidazole (MDZ), dimetridazole (DTZ), ornidazole (ODZ), chlo-ramphenicol (CAP), thiamphenicol (THI), florfenicol (FFC), sulfadiazine (SDZ) and sul-famethazine (SMZ) (Supplementary Figure S6), were subjected to fluorescence detection using the **RhB@Zr-Eddc**.

**FIGURE 2:**
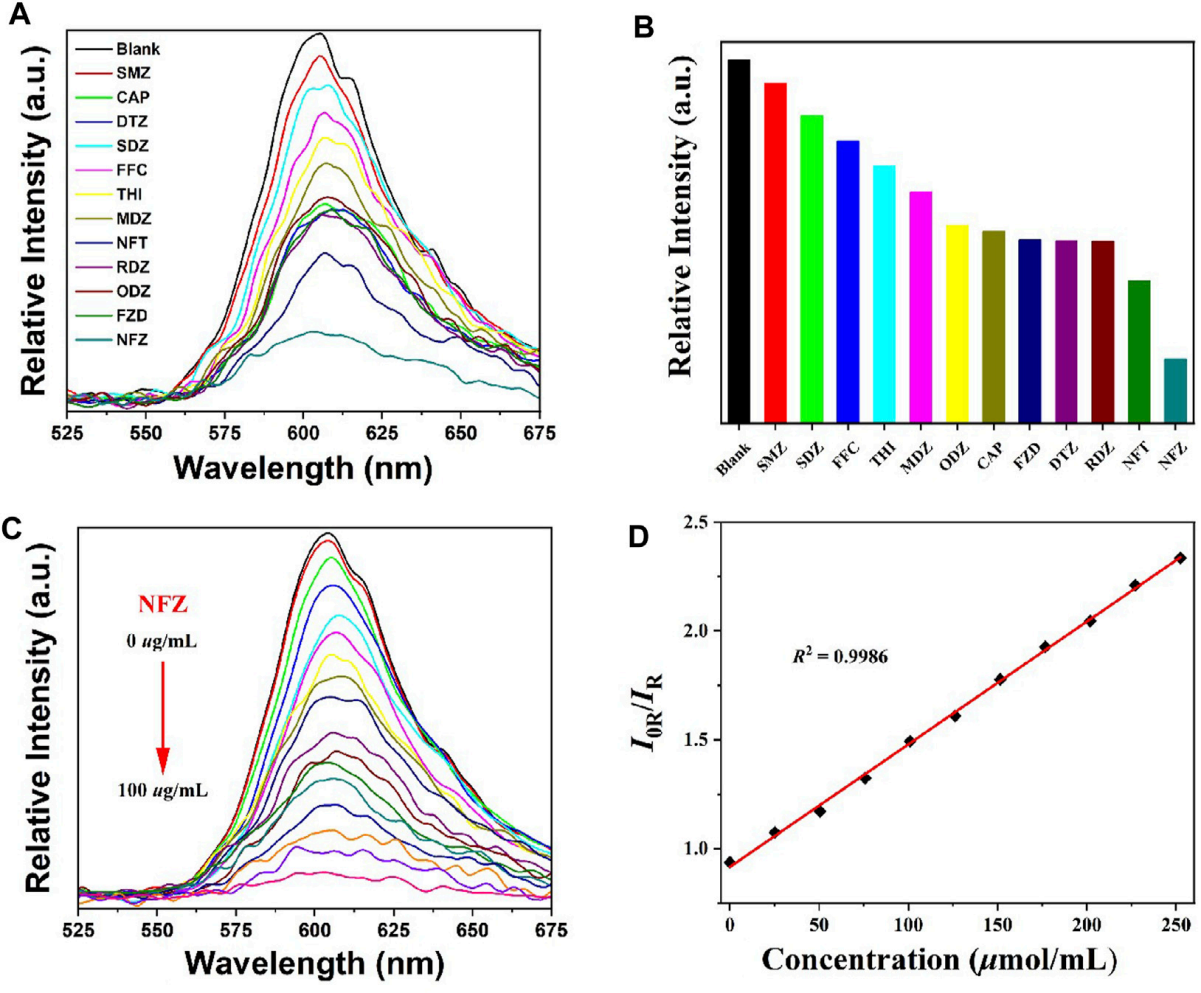
**(A)** and **(B)** The emission spectra and relatively intensity of **RhB@Zr-Eddc** with the different antibiotics; **(C)** Effects of different concentrations of NFZ on the **RhB@Zr-Eddc** fluorescence sensing; **(D)** The linear correlation of (I0/I) vs concentrations of NFZ antibiotic.

5 mg of **RhB@Zr-Eddc** was immersed in 2 mL of twelve different antibiotic aqueous solutions with a concentration of 10 *μ*g/mL. The mixtures underwent ultrasonic treatment to form suspensions, and their fluorescence spectra were determined under the same conditions. The experimental results revealed varying degrees of quenching effects of antibiotics on **RhB@Zr-Eddc** with the quenching effect ranking as follows: NFZ > NFT > RDZ > DTZ > FZD > CAP > ODZ > MDZ > THI > FFC > SDZ > SMZ. Notably, the antibiotic NFZ exhibited the most pronounced fluorescence quenching effect (Figures 2A,B).

To investigate the efficacy of **RhB@Zr-Eddc** as a fluorescent probe for the NFZ antibiotic, 5 mg of **RhB@Zr-Eddc** was immersed in 2 mL NFZ aqueous solutions with different concentrations, whose fluorescence spectra were determined under uniform conditions. As the concentration of NFZ antibiotic increased, the fluorescence intensity of **RhB@Zr-Eddc** was significantly reduced (Figure 2C). At a concentration of 100 *μ*g/mL NFZ, the fluorescence intensity of **RhB@Zr-Eddc** was nearly completely quenched. According to the Stern–Volmer equation, the quenching constants (*K*_sv_) value of the **RhB@Zr-Eddc** acted the fluorescent probe for detecting the NFZ antibiotic is 7.08 × 10^4^ M^-1^ with the range of 0–50 μg/mL (Figure 2D). In addition, according to the equation (LOD = 3*σ*/*K*_SV_, where *σ* is the standard deviation for eleven repeated luminescent measurements and *K*_sv_ is the quenching constants), the limits of detection (LODs) are 0.15 μM, indicating that **RhB@Zr-Eddc** demonstrates high detection performance for NFZ antibiotic compared with other MOFs (Supplementary Table S1).

To verify the high selectivity of **RhB@Zr-Eddc** for detecting NFZ antibiotic, 5 mg of **RhB@Zr-Eddc** was immersed in 2 mL solutions containing of NFZ antibiotic and interfering antibiotics with the concentration of 10 μg/mL, whose fluorescence spectra were then obtained under the same conditions. Experimental results showed a clear quenching effect on the emission intensities of **RhB@Zr-Eddc** with the addition of an equivalent amount of NFZ antibiotic (Supplementary Figure S7). This suggests that the emission intensities of **RhB@Zr-Eddc** can be rapidly quenched by NFZ antibiotic in the presence of eleven kinds of interfering antibiotics, indicating high selectivity and resistance to interference in the detection of NFZ antibiotic (Wang et al., 2016; Zhou et al., 2021; Wang et al., 2022; Li et al., 2023; Wang et al., 2023; Wen et al., 2023).

The sensing mechanism of **RhB@Zr-Eddc** for antibiotics can be attributed to the collision interaction between **RhB@Zr-Eddc** and antibiotic structures, and this interaction depletes energy transfer and resonance energy transfer (Wang et al., 2016; Cong et al., 2021; Lei et al., 2021; Rani et al., 2022; Su et al., 2022; Zhang et al., 2023; Fan et al., 2024). In summary, these findings indicate that **RhB@Zr-Eddc** demonstrates highly selective and sensitive detection capabilities for the NFZ antibiotic.

### 2.3 WLED device

**FIGURE 3:**
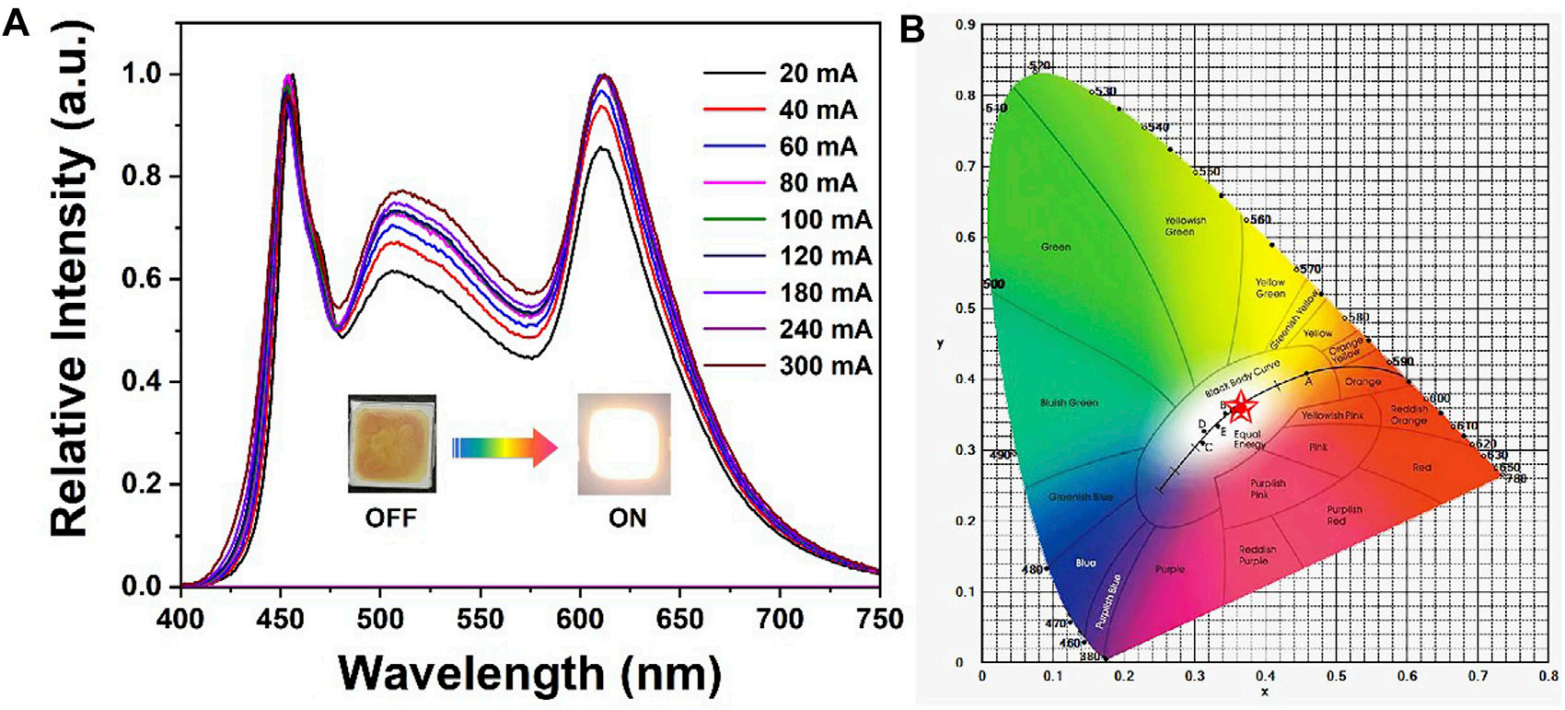
**(A)** and **(B)** The fluorescence emission spectra and CIE of WLED device based on the **RhB@Zr-Eddc** and commercial green phosphor with 455 nm blue LED chip.

Due to **RhB@Zr-Eddc** exhibits red-light emission, the prepared samples were utilized to create White Light Emitting Diode (WLED) devices aiming for excellent Correlated Color Temperature (CCT), Color Rendering Index (CRI), and Luminous Efficiency (LE) (Wen et al., 2017; Gutiérrez et al., 2020; Tang et al., 2020; Yuan et al., 2021; Atoini et al., 2023). The **RhB@Zr-Eddc** samples were combined with a commercial green phosphor (Y_3_Al_5_O_12_:Ce^3+^, Ga^3+^) in a mass ratio of 5: 1. The resulting composite phosphors were then applied to the curved surface of a commercial 455 nm blue LED chip to fabricate the WLED device. The WLED device exhibited peaks corresponding to the blue LED chip at 455 nm, the commercial green phosphor (Y_3_Al_5_O_12_:Ce^3+^, Ga^3+^) at 510 nm, and the **RhB@Zr-Eddc** at 612 nm (Figure 3A).

Furthermore, as the current increased from 20 mA to 300 mA, the Correlated Color Temperature (CCT) and Color Rendering Index (CRI) values underwent changes. The maximum values for CCT and CRI were determined to be 4710 K and 89.2, respectively (Table 1). Conversely, the Luminous Efficiency (LE) value exhibited a decrease as the current decreased from 20 mA to 300 mA, reaching its maximum value at 43.17 lm/w (Table 1). Additionally, the CIE values were modified from (0.355, 0.353) to (0.354, 0.357) (Figure 3B and Table 1), aligning closely with the standard white light emission values of (0.333, 0.333) (Liu et al., 2019; Karmakar and Li, 2022; Huang et al., 2023; Wu et al., 2024).

**TABLE 1:** The LE, CCT, CRI, and CIE Coordinates of the WLED device based on the **RhB@Zr-Eddc** and commercial green phosphor with 455 nm blue LED chip.

Current (mA)	LE (lm/W)	CCT (K)	CRI	CIE x	CIE y
20	43.17	4634	83.9	0.355	0.353
40	41.09	4541	85.2	0.358	0.356
60	38.44	4541	86.1	0.359	0.357
80	36.28	4541	86.8	0.359	0.358
100	34.36	4532	87.2	0.359	0.359
120	32.62	4521	87.5	0.359	0.359
180	28.27	4559	88.4	0.358	0.359
240	24.39	4627	89	0.356	0.358
300	21.12	4701	89.2	0.354	0.357

## 3 Materials and methods

### 3.1 Materials

All reagents were obtained commercially and used without further purification. Powder X-ray diffraction (XRD) patterns were recorded using a Desktop X-ray Diffractometer (Ultima-IV, Rigaku). Thermogravimetric experiments were conducted using a TGA/NETZSCH STA449C instrument, heated from 25°C–800°C with a heating rate of 10°C/min in a nitrogen stream. Scanning electron microscopy (JSM6700-F) was employed to characterize the morphology of the samples. Luminescent tests were performed using a PerkinElmer LS55 instrument. The White Light Emitting Diode (WLED) device was measured using a HAAS-2000 High Accuracy Array Spectroradiometer (Everfine) at room temperature under different current densities. The CIE data were computed with the GoCIE program and plotted on the 1931 CIE coordinate diagram.

### 3.2 Synthesis of RhB@Zr-Eddc

4,4′-Stilbenedicarboxylic acid (H_2_Eddc) (90 mg), ZrCl_4_ (70 mg), and rhodamine B (RhB) (100 mg) were combined in a 50 mL polytetrafluoroethylene reaction vessel. Subsequently, 20 mL *N, N′*-dimethylformamide (DMF) and 2 mL glacial acetic acid were added to the mixture. The resulting blend underwent ultrasonication and was then placed in an oven at 120°C for 50 h. After completion, the reaction vessel was cooled to room temperature. The obtained product was washed three times with DMF and methanol, separated through centrifugation, and dried at 85°C to obtain the **RhB@Zr-Eddc**.

### 3.3 Fluorescence sensing

To assess the interaction between **RhB@Zr-Eddc** and antibiotics, 5 mg of **RhB@Zr-Eddc** was immersed in 2 mL of twelve different antibiotic aqueous solutions, each with a concentration of 10 μg/mL. The mixtures were subjected to ultrasonic treatment under 20 min to form suspensions, and their fluorescence spectra were determined under the same conditions. Moreover, to specifically explore the effect of nitrofurazone, 5 mg of **RhB@Zr-Eddc** was immersed in 2 mL of nitrofurazone aqueous solutions with varying concentrations, and their fluorescence spectra were determined under the same conditions. To verify the selectivity of **RhB@Zr-Eddc** for the detecting NFZ antibiotic, 5 mg of **RhB@Zr-Eddc** was immersed in 2 mL solutions containing of NFZ antibiotic and interfering antibiotics with the concentration of 10 μg/mL and the volume ratio was 1:1, whose fluorescence spectra were then obtained under the same conditions.

### 3.4 WLED Encapsulation and determination experiment

The synthesis of White Light Emitting Diode (WLED) devices involved combining the as-synthesized **RhB@Zr-Eddc** and commercial green phosphor (Y_3_Al_5_O_12_:Ce^3+^, Ga^3+^) in a mass ratio of 5: 1. This mixture was then combined with silica gel (Dow Corning, OE6550 A/B) in a powder to glue mass ratio of 1: 1. The resulting composite acted as the phosphor material applied to the curved surface of a commercial 455 nm blue LED chip to create the WLED devices. Subsequently, the WLED devices were heated at 150°C for 2 h. The capabilities of the LED devices were then measured at room temperature under different current densities using the HAAS-2000.

## 4 Conclusion

Highly thermal stable **RhB@Zr-Eddc** was synthesized using a one-pot reaction method under hydrothermal conditions. The material was characterized through XRD, SEM, and TGA. **RhB@Zr-Eddc** demonstrated exceptional thermal stability, enduring temperatures up to 550°C, which also emitted a bright red-light at 605 nm and exhibited high selectivity in detecting nitrofurazone (NFZ) among eleven antibiotics. Additionally, **RhB@Zr-Eddc** was combined with commercial green phosphor powders and encapsulated onto a 455 nm blue LED chip, resulting in an excellent White Light Emitting Diode (WLED) device. The WLED device exhibited outstanding Correlated Color Temperature (CCT), Color Rendering Index (CRI), and Luminous Efficiency (LE). This suggests that **RhB@Zr-Eddc** holds potential for use in WLED devices, offering highly selective and sensitive detection capabilities for nitrofurazone antibiotics in future applications.

## Data Availability

The original contributions presented in the study are included in the article/Supplementary Material, further inquiries can be directed to the corresponding authors.
