# Supplementary material for: Highly thermostable RhB@Zr-Eddc for the selective sensing of nitrofurazone and efficient white light emitting diode
Source: Front Chem. 2024 Aug 2;12:1444036. doi: 10.3389/fchem.2024.1444036 (PMC11327442; doi:10.3389/fchem.2024.1444036)
Supplement: Supplementary file 1 [file DataSheet1.docx]

Supplementary Material

Highly Thermostable RhB@Zr-Eddc for the Selective Sensing of Nitrofurazone and Efficient White Light Emitting Diode

Yanqiong Shen ^1^, Di Ma ^1^, Mian Zhao ^2,*^, Jinjie Qian ^3,*^ and Qipeng Li ^1,*^

^1^ College of Chemistry and Chemical Engineering, Zhaotong University, Zhaotong, 657000, P. R. China

^2^ Experimental Center for Teaching, Hebei Medical University, Shijiazhuang, 050017, P. R. China;

^3^ College of Chemistry and Materials Engineering, Wenzhou University, Wenzhou, 325035, P. R. China

***Correspondence:** 18601529@hebmu.edu.cn, jinjieqian@wzu.edu.cn and qpli@ztu.edu.cn


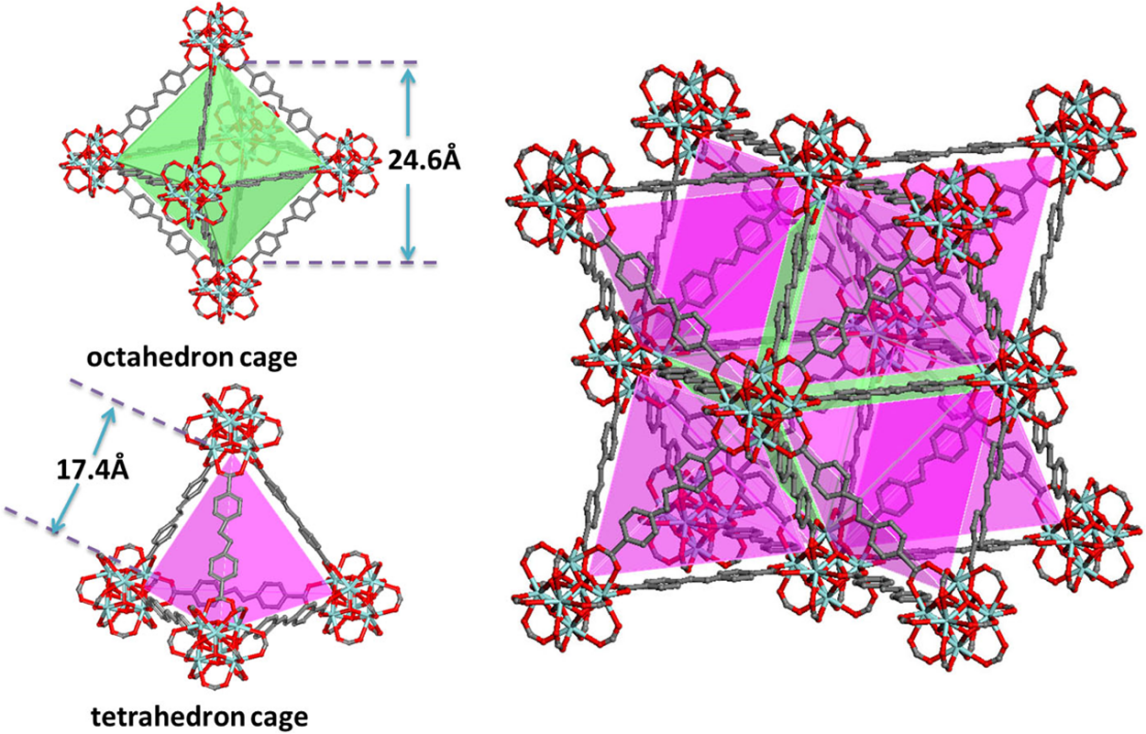


**Figure S1** Structural diagram of the **Zr-Eddc**.


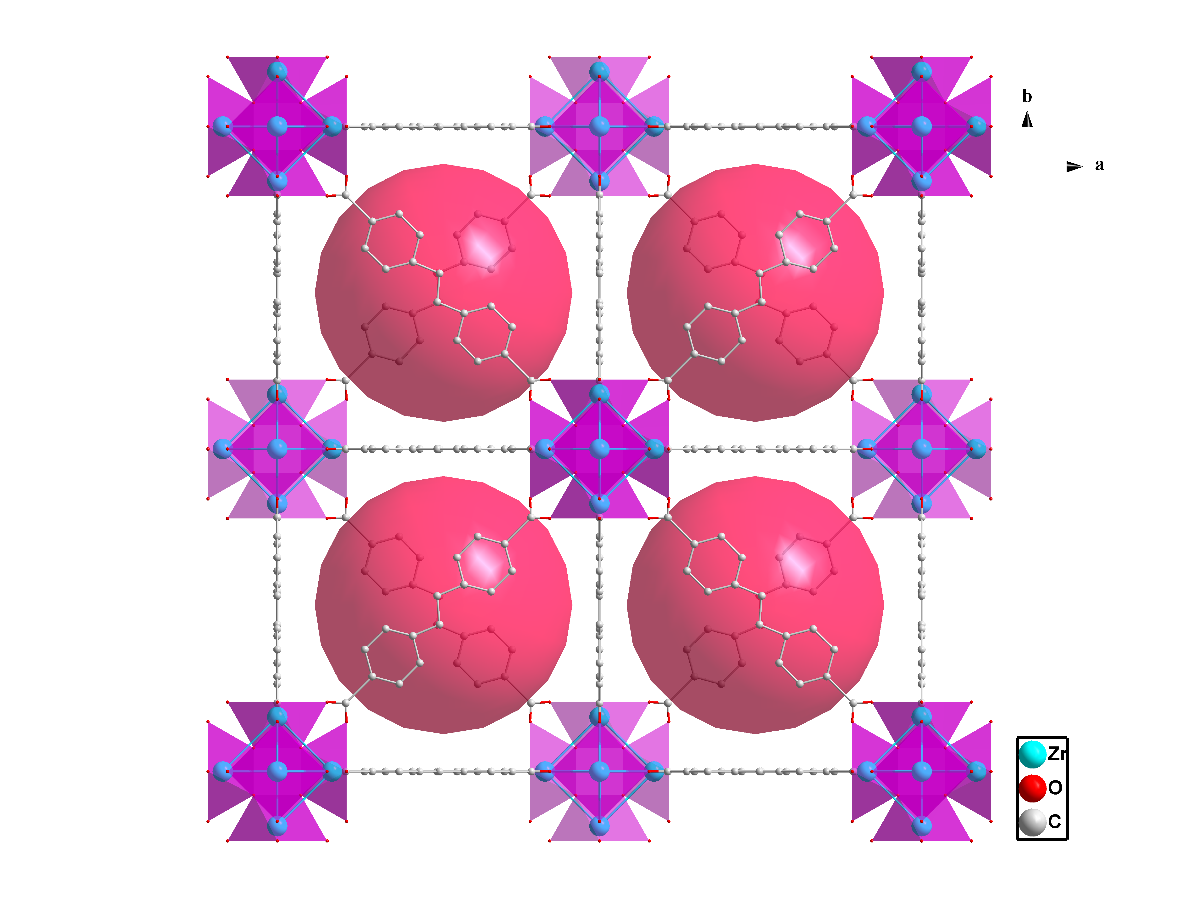


**Figure S2** Structural diagram of the **RhB@Zr-Eddc**.

**Figure S3** Chemical structures of the **RhB**.


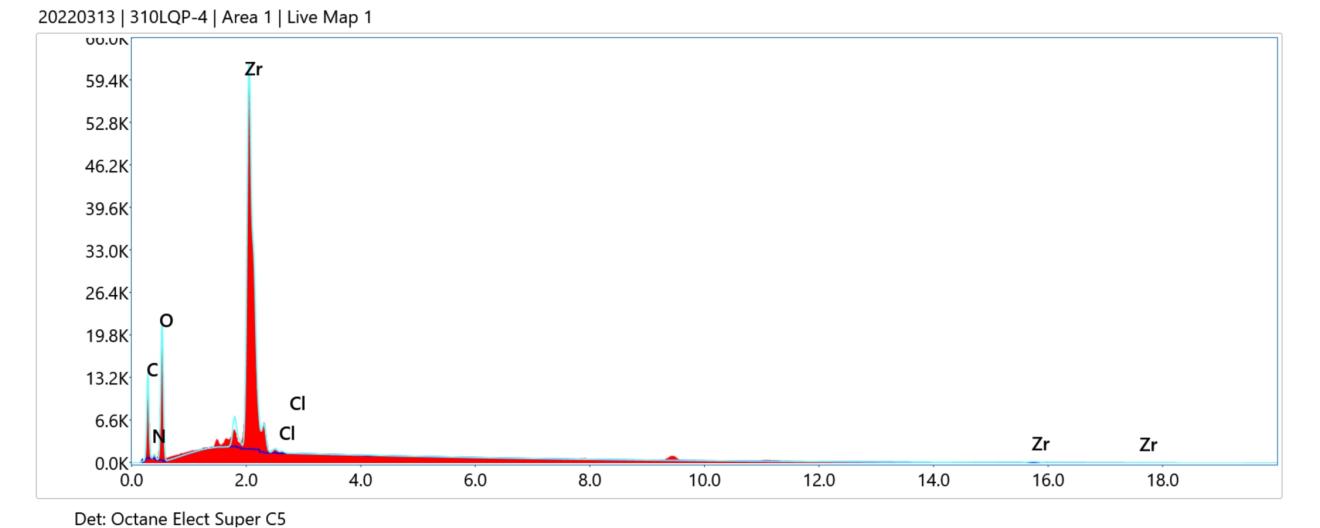


**Figure S4** The EDS energy map of **RhB@Zr-Eddc**.


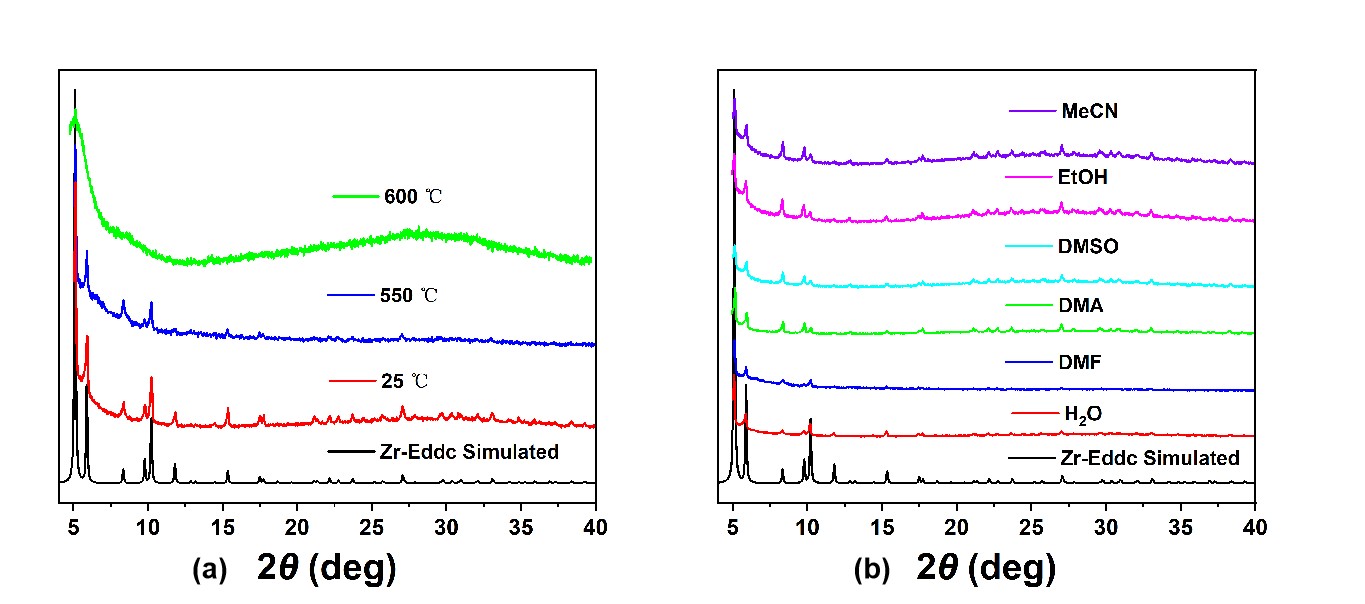


**Figure S5** (a) The Variable temperature XRD of **RhB@Zr-Eddc** and the XRD of **RhB@Zr-Eddc** in different solvents.

**
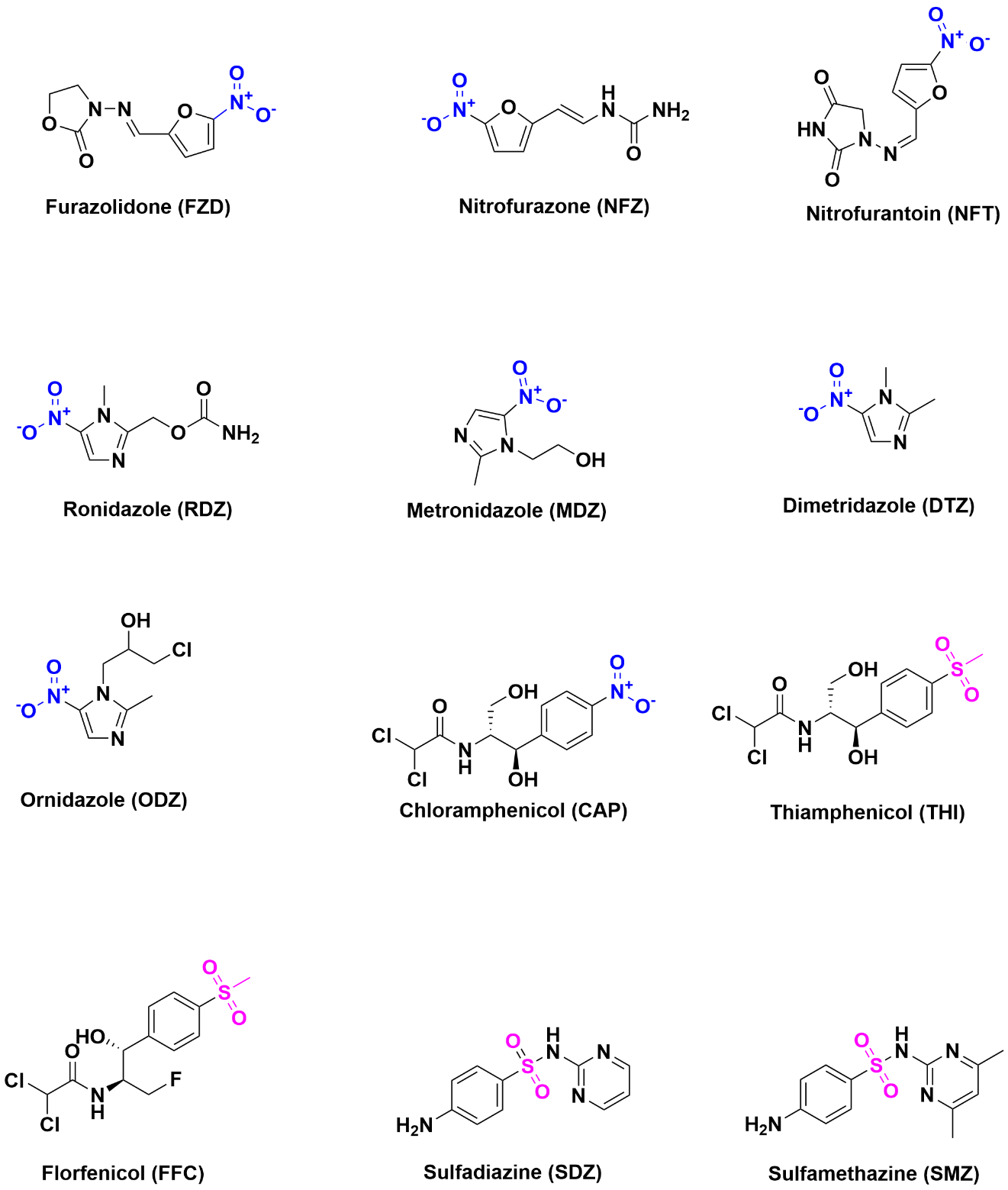
**

**Figure S6** Chemical structures of the twelve antibiotics.

**
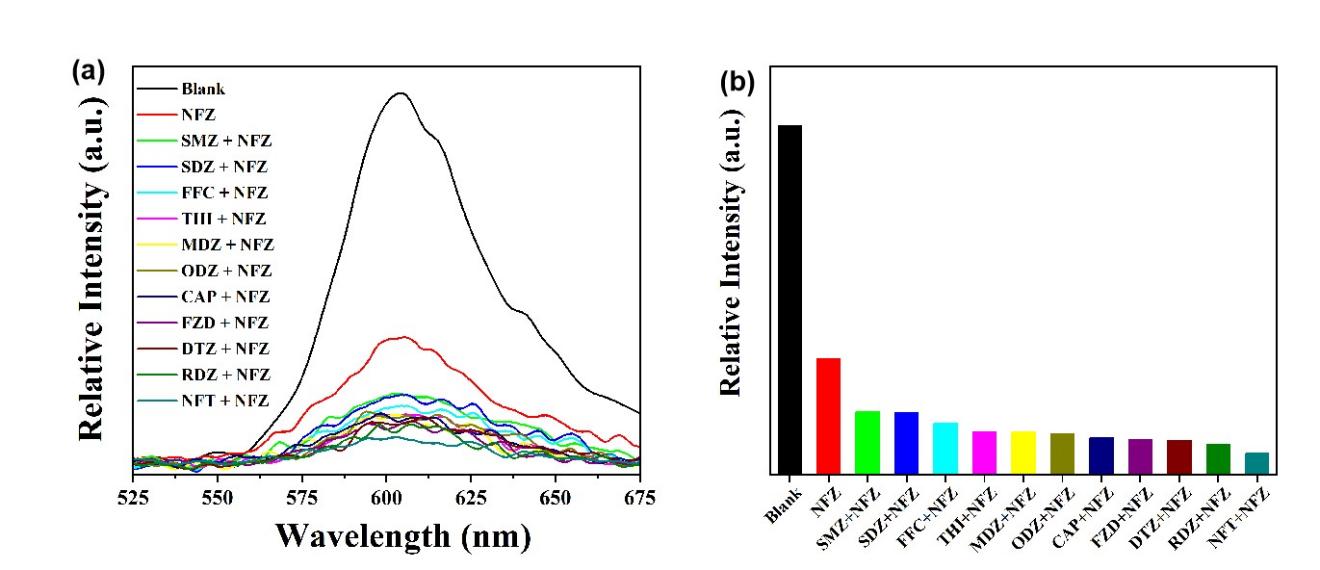
**

**Figure S7** The relatively intensity of **RhB@Zr-Eddc** with the selectively sensing of NFZ antibiotic.

**Table S1** Detection performances and K_sv_, media and LODs of MOF-based fluorescence sensors for NFZ

| **MOFs** | **Ksv** | **LODs** | **Refs** |
| --- | --- | --- | --- |
| V102 | 6.38 × 10^4^ M^-1^ | 0.2 ppm | S1 |
| [Ln(Hpta)(C_2_O_4_)]·3H_2_O | 1.1 × 10^4^ M^-1^ | 8.1×10^-5^ M | S2 |
| [Cd_10_(DDB)_4_(bpz)_8_] | 1.05 × 10^5^ M^-1^ | 0.03 ppm | S3 |
| [Zn(tptc)_0.5_(bimb)] | 9.556 × 10^4^ M^-1^ | 0.03 ppm | S3 |
| [Zn(NH_2_-TCB)] | 4.85 × 10^4^ M^-1^ | 0.06 ppm | S3 |
| ZnHoMOF | 5.406 × 10^4^ M^-1^ | 0.05 ppm | S3 |
| BUT-12 | 1.1 × 10^5^ M^-1^ | 58 ppb | S4 |
| BUT-13 | 7.5 × 10^4^ M^-1^ | 90 ppb | S4 |
| Zn-CPTA | 1.02 × 10^5^ M^-1^ | 0.126 *μ*M | S5 |
| Zr_6_O_6_(OH)_2_(TDCA)_4_(CH_3_COO)_2_ | -- | 2.50 × 10^-7^ M | S6 |
| [Cd_2_(L)(TDCA)_2_(H_2_O)_2_·0.5C_2_H_3_N]_n_ | 3.85 × 10^3^ M^-1^ | 2.02 × 10^-6^ M | S6 |
| [Cd(mba)(bpdb)_0.5_] | 8.1498 × 10^4^ M^-1^ | 0.123 μM | S7 |
| {[Cd_3_(L)_2_(bbi)_2_(H_2_O)_2_]·2H_2_O} | 8.26 × 10^3^ M^-1^ | 1.83 ppm | S8 |
| UiO-66-NH_2_ | -- | 5.5 × 10^-9^ M | S9 |
| {[Cd(BTA)_0.5_(L)_0.5_]_n_} | 4.5 × 10^−7^ M^-1^ | 1.4 × 10^-5^ M | S10 |
| {[Cd(HBTC)(L)]_n_} | 9.0 × 10^−7^ M^-1^ | 6.2 × 10^-6^ M | S10 |
| Zn-MOF | 1 × 10^4^ M^-1^ | 0.69 *u*M | S11 |
| **FCS-4** | 11352 M^−1^ | 0.73 ppm | S12 |
| **FCS-5** | 37448 M^−1^ | 0.22 ppm | S12 |
| **RhB@Zr-Eddc** | **7.08×10^4^ M^-1^** | **0.15 *u*M** | **This work** |

“-”represent the data are not listed.

1. Hou. S. L.; Dong. J.; Jiang. X. L.; Jiao. Z. H.; Wang. C. M.; Zhao. B.; Interpenetration-dependent luminescent probe in indium organic frameworks for selectively detecting nitrofurazone in water, *Anal. Chem.,* **2018**, 90, 1516-1519.
2. Duan. L. J.; Zhang. C. C.; Cen. P. P.; Jin. X. Y.; Liang. C.; Yang. J. H.; Liu. X. Y.; Stable Ln-MOFs as multi-responsive photoluminescence sensors for the sensitive sensing of Fe^3+^, Cr_2_O_7_^2−^, and nitrofuran. *CrystEngComm.,* **2020**, 22, 1695-1704.
3. Yin, J.; Li, W.; Li, W.; Liu, L.; Zhao, D.; Liu, X.; Hu, T.; Fan, L. Heterometallic ZnHoMOF as a Dual-Responsive Luminescence Sensor for Efficient Detection of Hippuric Acid Biomarker and Nitrofuran Antibiotics. *Molecules.,* **2023**, 28, 6274.
4. Wang. B.; Lv. X. L.; Feng. D. W.; Xie. L. H.; Zhang. J.; Li. M.; Xie. Y. B.; Li. J. R.; Zhou. C. Z.; Highly stable Zr(IV)-based metal-organic frameworks for the detection and removal of antibiotics and organic explosives in water, *J. Am. Chem. Soc.,* **2016**, 138(19): 6204-6216.
5. Wang, W. Z.; Yang, F.; Yang, Y. C.; Wang, Y. Y.; Liu, B.; Rational Synthesis of a Stable Rod MOF for Ultrasensitive Detection of Nitenpyram and Nitrofurazone in Natural Water Systems, *J. Agric. Food Chem.,* **2022**, 70, 15682-15692.
6. Wen, M. Y.; Fu, L. S.; Dong, G. Y.; Two Cd(II)-MOFs containing pyridylbenzimidazole ligands as fluorescence sensors for sensing enrofloxacin, nitrofurazone and Fe^3+^, *Journal of Molecular Structure.,* **2023**, 1285, 135488.
7. Wang, K. M.; Bai, X. L.; Zhao, X.; Dong, Y. Q.; Zhao, R. T.; Zhou, J.; Yu, H. M.; Li, L. F.; Tang, H. J.; Ma, Y. L.; Highly sensitive fluorescence detection of nitrofurazone and nitrofurantoin in milk and honey using a hydrostable Cd(Ⅱ) metal-organic framework, *Journal of Molecular Structure.,* **2023**, 1295, 136114.
8. Zhou, S. H.; Lu, L.; Liu, D.; Wang, J.; Sakiyama, H.; Muddassir, M.; Ejhieh, A. N.; Liu, J. Q.; Series of highly stable Cd(ii)-based MOFs as sensitive and selective sensors for detection of nitrofuran antibiotic, *CrystEngComm.,* **2021**, 23, 8043-8052.
9. Rani, R.; Deep, A.; Mizaikoff, B.; Singh, S.; Zirconium metal organic framework based opto-electrochemical sensor for nitrofurazone detection, *Journal of Electroanalytical Chemistry.,* **2022**, 909, 116124.
10. Fan, X. F.; Deng, X. C.; Cao, Q. L.; Dong, G. Y.; Fu, L. S.; Two robust dual-functional Cd(II) MOFs as luminescent sensors for quantitatively detection of nitrofurazone and Fe^3+^ ions, *Journal of Molecular Structure.,* **2024**, 1295, 136757.
11. Cong, Z. Z.; Song, Z. F.; Ma, Y. X.; Zhu, M. C.; Zhang, Y.; Wu, S. Y.; Gao, E. J.; Highly Emissive Metal-Organic Frameworks for Sensitive and Selective Detection of Nitrofuran and Quinolone Antibiotics, *Chem-an Asian Journal.,* **2021**, 16, 1773-1779.
12. Lei, Z.; Hu, L.; Yu, Z. H.; Yao, Q. Y.; Chen, X.; Li, H.; Liu, R. M.; Li, C. P.; Zhu, X. D.; Ancillary ligand enabled structural and fluorescence diversity in metal-organic frameworks: application for the ultra-sensitive detection of nitrofuran antibiotics, *Inorg. Chem. Front.,* **2021**, 8, 1290-1296.
